# A FPGA-Based, Granularity-Variable Neuromorphic Processor and Its Application in a MIMO Real-Time Control System

**DOI:** 10.3390/s17091941

**Published:** 2017-08-23

**Authors:** Zhen Zhang, Cheng Ma, Rong Zhu

**Affiliations:** Department of Precision Instrument, Tsinghua University, Beijing 100084, China; zhangz14@mails.tsinghua.edu.cn (Z.Z.); zr_gloria@mail.tsinghua.edu.cn (R.Z.)

**Keywords:** artificial neural networks, FPGA, neuromorphic processor, granularity variable, MIMO control

## Abstract

Artificial Neural Networks (ANNs), including Deep Neural Networks (DNNs), have become the state-of-the-art methods in machine learning and achieved amazing success in speech recognition, visual object recognition, and many other domains. There are several hardware platforms for developing accelerated implementation of ANN models. Since Field Programmable Gate Array (FPGA) architectures are flexible and can provide high performance per watt of power consumption, they have drawn a number of applications from scientists. In this paper, we propose a FPGA-based, granularity-variable neuromorphic processor (FBGVNP). The traits of FBGVNP can be summarized as granularity variability, scalability, integrated computing, and addressing ability: first, the number of neurons is variable rather than constant in one core; second, the multi-core network scale can be extended in various forms; third, the neuron addressing and computing processes are executed simultaneously. These make the processor more flexible and better suited for different applications. Moreover, a neural network-based controller is mapped to FBGVNP and applied in a multi-input, multi-output, (MIMO) real-time, temperature-sensing and control system. Experiments validate the effectiveness of the neuromorphic processor. The FBGVNP provides a new scheme for building ANNs, which is flexible, highly energy-efficient, and can be applied in many areas.

## 1. Introduction

In recent years, machine learning has entered into our daily life. When we communicate with smart phones using natural language or get pictures on digital cameras using face detection, artificial intelligence plays a key role in the process [1]. Over the past decade, Artificial Neural Networks (ANNs), including Deep Neural Networks (DNNs), have become the state-of-the-art methods and achieved amazing success in machine learning, especially in visual recognition, speech recognition, and other domains [2,3,4,5,6,7]. With significantly higher accuracy than traditional algorithms in various tasks like face recognition and image processing [8,9], DNNs have attracted the enthusiastic interest of internet giants such as Google [10,11], Microsoft [12], Facebook [13], and Baidu [14]. There are several hardware platforms for developing accelerated implementation of DNN models, including multicore CPUs [15], General Purpose Graphics Processing Units (GPGPUs) [16], Application Specific Integrated Circuits (ASICs) [17], and Field Programmable Gate Arrays (FPGAs) [18].

CPUs and GPUs are parts of General Purpose Processors (GPPs). The classic platforms based on CPU and GPU are SpiNNaker and Carlsim, correspondingly. The SpiNNaker machine is a specifically designed computer for supporting the sorts of communication found in the brain. It is based on the connection of processing nodes, which have eighteen ARM processor cores in one node. Over hundred neurons can be modelled in each processor core and there are one thousand input synapses connected to each neuron [15]. The Carlsim is a GPU-accelerated simulator which is capable of simulating the neural model [19]. GPPs can provide a high degree of flexibility and tend to be more readily accessible. However, the hardware performs with less energy efficiency, which is of particular importance in embedded, resource-limited applications or server-based large scale deployments [1].

Recently, the development of the neuromorphic processor has received increasing attention. For GPPs, application level execution relies on the traditional von Neumann architecture. It stores instructions and data in external memory to be fetched. The von Neumann architecture is non-scalable and inefficient in executing massive neural networks, and the von Neumann bottleneck can be mitigated by colocated computation and memory [17].

**Figure 1:**
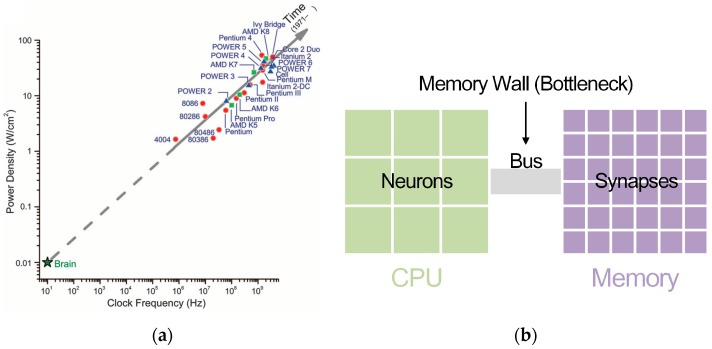
Von Neumann architecture chip features. (**a**) Comparison of power densities and clock frequencies between brain and von Neumann architecture processors. Reprinted with permission from AAAS [17]. (**b**) Schematic diagram of implementing neural networks in von Neumann architecture computers.

As seen in Figure 1, the centralized sequential von Neumann architecture computer is different from the brain’s distributed parallel architecture. The processor’s increasing clock frequencies and power densities are headed away from the operating point of the brain. As to implementing neural networks in a von Neumann architecture computer, a central processor has to simulate communication infrastructure and a great number of neurons. The bottleneck which serves as the communication channel between the processor and external memory causes power-hungry data movement while retrieving synapse states and updating neuron states [17]. A single processor is not suitable for simulating highly interconnected networks, which will cause interprocessor messaging explosions [17].

In comparison, the neuromorphic processor has a different architecture. The special computation structure of neural networks implies that the hardware suitable for exploiting pipeline parallelism takes advantage. When GPPs execute a parallel based on multiple cores, specially designed ASICs and FPGAs can support inherently pipelined and multithreaded applications, which are not based on the von Neumann architecture. They have the ability to exploit the large extent of pipeline parallelism and distributed on-chip memory. Similar to the brain, the neuromorphic processor has distributed and integrated computation and memory, and operate in parallel [1,20]. Developing the neuromorphic processor via the ASIC-based or FPGA-based approach shows their different advantages.

ASICs are dedicated to a specific application. In recent years, the TrueNorth chip, which is developed by IBM, has attracted considerable attention. It is a low power, high parallel chip with 4096 neurosynaptic cores. The core is the basic block, which has a crossbar array for synaptic connections and neurons for calculation. Each core contains 256 input axons, a 64k synaptic crossbar, and 256 neurons. The TrueNorth chip is a neurosynaptic chip produced via a standard-CMOS manufacturing process [17,20]. Generally, the ASICs can provide high performance. At the same time, they are expensive and time consuming to produce and the architectures are relatively fixed and inflexible [1].

Traditionally, we must consider the flexibility, performance, and energy efficiency when evaluating hardware platforms. On the one hand, GPPs can be highly flexible and easy to use, but perform relatively inefficiently. On the other hand, ASICs work with high efficiency at the cost of being inflexible and difficult to produce [1]. As a compromise, the FPGA-based approach has drawn a significant number of applications from scientists and become one of the most promising alternatives, due to its low power usage, high performance, reprogrammability, and fast development round [21,22,23,24,25,26]. FPGAs often provide better performance per watt than GPPs and naturally fit with the neural network execution [1]. Table 1 shows a comparison of neuromorphic processors and GPPs mentioned above for implementing the neural network. Microcontroller Unit (MCU), which serves as a kind of low cost GPP, is also included for full comparison.

In this paper we propose a FPGA-based granularity variable neuromorphic processor (FBGVNP) with integrated computing and addressing cells. The presented neuromorphic processor consists of neuromorphic cores structured by a router, a neural computing unit, and a data-transmission controller. The router is used to build connections and keep communications between different cores. Meanwhile, the neural computing unit is composed of neuron computing cells, which can process computing and addressing simultaneously. Moreover, the data-transmission controller is responsible for the computing result transmissions. The traits of the neuromorphic processor can be summarized as follows.

(1) *Granularity variability*: The number of the cells in one neural computing unit can vary, which will enhance the flexibility of the neuromorphic core compared with fixed architectures. The neuron computing cells perform as the basic elements in this architecture. One can expand the size of the cells as required. That will make the core better suited for different applications.
(2) *Scalability*: The scalability is achieved by connecting different cores with routers and extending inner neural computing units. The data interaction through routers links the neuromorphic cores so they become whole. Generally, routers serve as the communication nodes in the multi-core network and the network scale can be extended as needed.
(3) *Integrated computing and addressing ability*: The neuron computing cell combines together computing and addressing abilities. The data transmission uses the broadcast mechanism. On this basis, a neuron computing cell serves as a data receiving and processing terminal and the two processes are executed simultaneously, which makes the computations perform in parallel.

The paper is organized as follows. Section 2 introduces the architecture of the FBGVNP. In Section 3, a neural network is mapped to the presented neuromorphic processor and applied in a multi-input multi-output (MIMO) temperature sensing and control test platform. Then, the experiment results and discussions are given. Finally, the conclusions are drawn in Section 4.

## 2. Neuromorphic Processor Architecture 

### 2.1. Architecture of FBGVNP 

**Figure 2:**
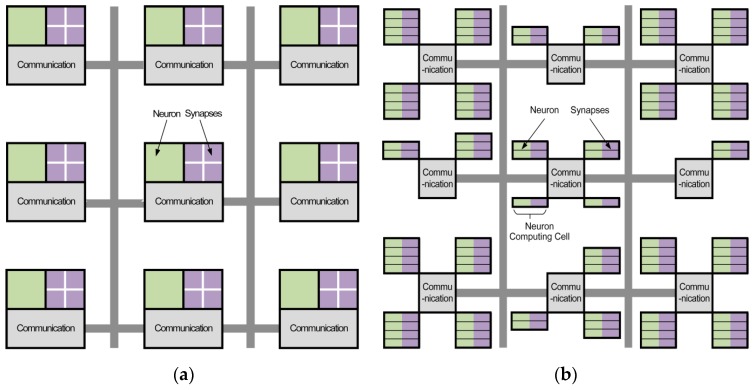
Conceptual blueprint comparison of TureNorth and FPGA-based, granularity-variable neuromorphic processor FBGVNP. (**a**) Conceptual blueprint of TureNorth. (**b**) Conceptual blueprint of FBGVNP.

The architecture of the processor is designed to be granularity variable, scalable, and parallel, with a router, a neural computing unit, and a data-transmission controller as the basic blocks. The neural computing unit consists of neuron computing cells, which can conduct computing and addressing simultaneously. The comparison of TrueNorth and FBGVNP is shown in Figure 2.

**Figure 3:**
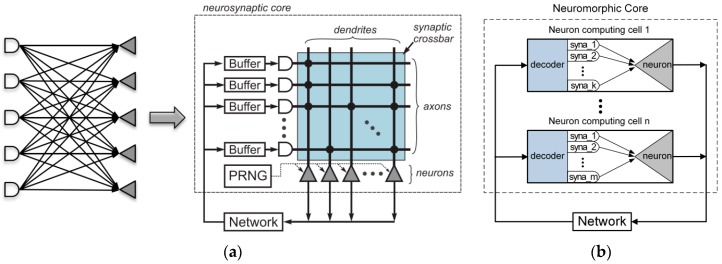
Structur diagram comparison of TureNorth and FBGVNP. (**a**) Bipartite graph (left) and logical representation (right) of a TrueNorth core. © [2015] IEEE. Reprinted, with permission, from [20] (**b**) Logical representation of a FBGVNP core.

From a structural view, the link relationship of TureNorth is shown in Figure 2a. The structure of TureNorth core can be divided to three parts. The communication module is used to keep the data interaction via a connected network. The synapses module is responsible for connecting different neurons, while the neuron module serves as a neural computation center. The synapses module adopts crossbar architecture, which makes the number of neurons in one core fixed at 256 [20]. Its basic block is a core which contains a neural network with 256 inputs and 256 outputs connected through 256-by-256 synaptic connections [17]. As shown in Figure 3a, a TureNorth neurosynaptic core is composed of input buffers, axons, dendrites, and neurons. The horizontal and vertical lines represent axons and dendrites correspondingly. Also, the triangles represent neurons. The block dots, which serve as synapses, represent the connections between axons and dendrites [20]. A synaptic crossbar organizes the synapses in each core. The neuron’s output is connected to the axon’s input located in the same core or in a different core over the routing network.

Figure 2b shows the connecting architecture of FBGVNP. In FBGVNP, the neuron and its corresponding synapses are mixed in one computing cell, which serves as the basic element. The contents of the synapses vary in different computing cells. A communication module connects several neural computing units in one core, while the number of neurons can change in one core as needed. Building on the local, one can link multiple cores together by distributed global connectivity and construct more complex networks. Unlike TrueNorth, the basic structure of FBGVNP is the neuron computing cell, and the number of the cells can change in a core as needed. In one cell, the neuron and its corresponding synapses are mixed together.

The structure of a neuromorphic core in FBGVNP can be seen in Figure 3b. The neuron computing cell serves as the basic element. It consists of two parts: decoder and neuron. The data transmission uses broadcast mechanism. The neuron outputs (axons) are passed to the inputs of the neuron computing cells in a sequence via the routing network. Each neuron can send out an axon packet to a target in the same local core or another external core. Then, the decoders in the target core receive the past axon packet and start to decode. There are different pre-stored dendrite records in each decoder, and the decoded axon information will be compared with the local dendrite records. After that, the valid axon information will be transferred to the neuron for computing. Therefore, axonal branching is executed hierarchically in two steps. Firstly, a connection passes through a long distance between the cores. Secondly, via a short distance within a core, it is broadcasted to multiple neuron computing cells after arriving at its target core.

### 2.2. FBGVNP Internals

**Figure 4:**
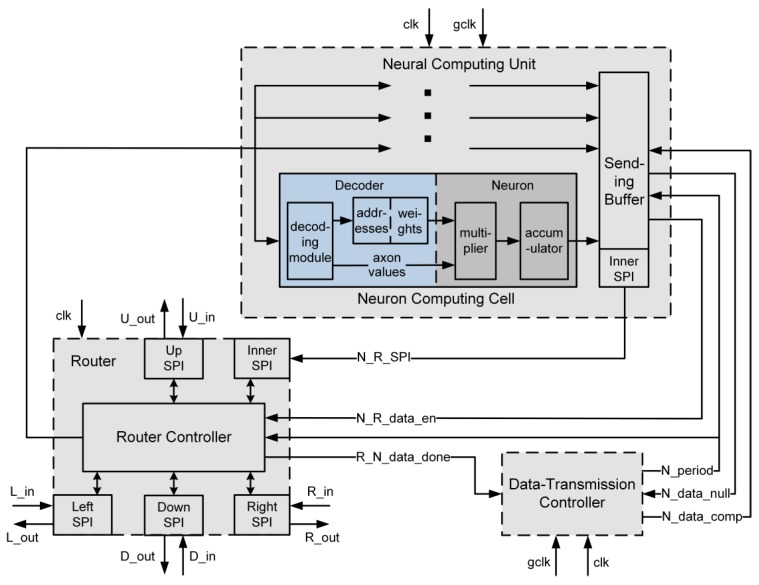
Internal structure of the FBGVNP core composed of router, neural computing unit, and data-transmission controller. clk: clock. gclk: global clock.

The FBGVNP core is composed of a router, a neural computing unit, and a data-transmission controller. The details of the neuromorphic core can be seen in Figure 4. The router is used for communicating with other cores, and the neural computing unit is where the neuron connection and computing take place. In addition, the data-transmission controller is used for transmitting neuron outputs.

The FBGVNP architecture supports the interconnected neural network by building a network of the presented neuromorphic cores. Connections between cores are implemented by sending data though the router network. As shown in Figure 4, in a one router, one neural computing unit structure, each router is decomposed into five separated data transfer directions: (1) left; (2) right; (3) up; (4) down; and (5) local. Five input ports exist to receive the routing packets from local core or nearest-neighbor routers in the left, right, up, and down directions. In addition, there are also five output ports corresponding. The transmitted data packet between routers has the following format as shown in Table 2, and the data length of each part can be changed as needed.

Upon received a packet, the information in the dx or dy fields (number of hops in the left/right or up/down directions correspondingly) is used to pass the packet out to one of five destinations: the local neural computing unit or nearest neighbor routers in four different directions. For using the X-Y dimension order routing strategy, the dx field is decremented to right hop or incremented to left hop, and packets are routed in the horizontal direction first. When dx becomes 0, the dy field is decremented to above hop or incremented to below hop. When the dx and dy both become 0, the destination unit index field, which stores the target neural computing unit address, will be resolved. Then, after stripping the associated bits, the remaining 16 bits are sent to the target neural computing unit. The destination cell index field denotes the address of the outputting neuron, which can be matched by the neurons in the target core. Thus, within a routing window, a packet from any core can be sent to a neuron at any destination core, and the axon value is stored in the neuron output field.

The neural computing unit is where the neuron connection and the computing take place. The packet transferred from the router will be broadcasted to all the cells in the neural computing unit. There are different address-weight pairs pre-storage in the decoders. After the decoding of the packet in each cell, the neuron output field data (axon value) of the address-matched packet will be sent to the neuron as well as the corresponding pre-storage weight. In the neuron, the axon value and weight will be multiplied and then sent to an accumulator. The result of the accumulator will become the neuron output.

The data-transmission controller serves as the scheduler in computing the result transmissions. It is used to control the processes of outputting neuron computing results stored in the sending buffer.

Comparably, the number of the neural computing units connected to a router can be extended in a similar way.

The data transmission of FBGVNP proceeds according to the following steps.

(1) A neuromorphic core receives a packet from the network and resolves it. If not equal to zero, the dx field will be decremented or incremented and the packet will be sent to the corresponding right or left routing hop. Then, when dx becomes 0, the dy field will also be decremented or incremented in a vertical direction transmission until it turns to 0.
(2) The destination unit index field will be resolved to get the target unit neural computing unit address. The dx, dy, and destination unit index field bits are stripped and the remaining 16 bits are broadcasted to the cells in the target neural computing unit. The destination cell index field bits will be compared to the address entries pre-stored in each decoder. If they match, the corresponding weight in the decoder will be sent to the neuron for computing, as well as to the neuron output field bits in the packet. If not, the cell will work only when the next packet reaches the neuron.
(3) The neuron receives weight and neuron output field bits from the decoder and multiplies them. The result will be sent to an accumulator.
(4) When a synchronization trigger signal called the global clock arrives, each neuron outputs the accumulator result to the sending buffer and the core steps in the data sending process.
(5) After the global clock arrives, the core step is in the data transmission period. The data-transmission controller initializes the N_period signal. It is a number denoting which neuron’s computing result is selected to be transferred out. At the same time, the N_data_comp signal pulses once and drives a process verifying the validity of the selected neuron’s computing result stored in the sending buffer. If invalid, the N_data_null signal pluses once, and the core goes to another data transmission period. On the other hand, if valid, the N_R_data_en signal turns to the enabling state, until the end of this period’s data sending.
(6) When the N_R_data_en signal is enabled, the selected neuron’s computing result will be transferred to the router, if the inner serial peripheral interface (SPI) port’s buffer is not full. After the transferring, the router controller will send the packet targeting to the destination core and return back a R_N_data-done signal to the data-transmission controller. Then, the N_period and N_data_comp signals will be updated along with the core steps in another data transmission period.
(7) After the transferring of the last neuron’s computing result, the core will keep waiting for the next arrival of the global clock.

### 2.3. Features Comparison of FBGVNP and TrueNorth 

FBGVNP and TrueNorth both serve as a neuromorphic processor but have their respective features. The comparison of FBGVNP and TrueNorth can be seen in Table 3.

The main difference between FBGVNP and TrueNorth is from their corresponding structure, since FBGVNP is developed based on a reprogrammable FPGA, while TrueNorth uses a relatively fixed crossbar structure and is developed via ASIC-based approach. As mentioned above, ASIC-based TrueNorth has the highest energy efficiency.

**Figure 5:**
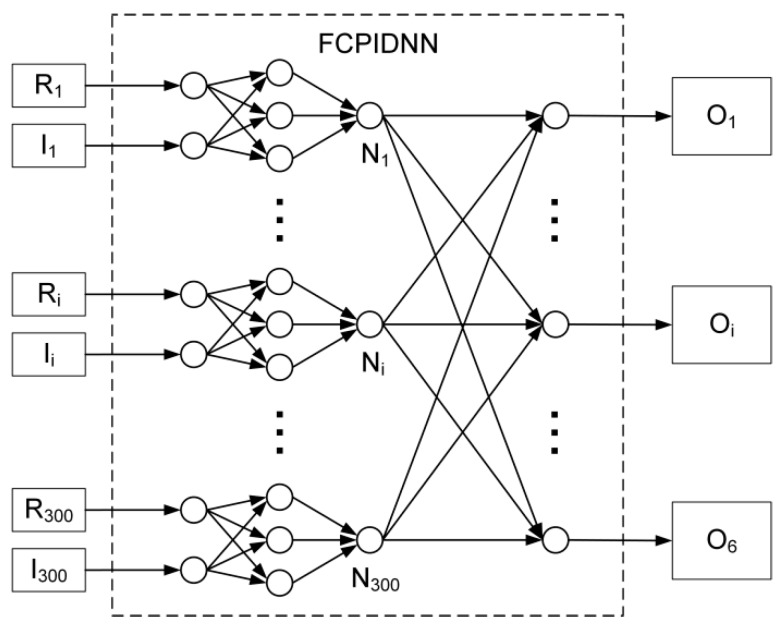
Structure diagram of the reference fully-connected, proportional–integral–derivative neural network (FCPIDNN).

Further, an execution comparison between the two processors based on a fully-connected, proportional–integral–derivative (PID) neural network (FCPIDNN) is presented as follows. The structure of the reference FCPIDNN is shown in Figure 5.

The FCPIDNN is a structure suitable for resolving multi-input, multi-output (MIMO), real-time control problems [27,28]. It inputs reference and sampled real signals, and outputs control signals to the actuators. The scale of the FCPIDNN depends on the number of input and output channels. The presented FCPIDNN in Figure 5 adopts a 300 inputs and 6 outputs configuration. The execution time comparison of FBGVNP and TrueNorth, based on the reference FCPIDNN, is presented in Table 4.

It is obvious to see that, based on the FCPIDNN, the execution time of the TrueNorth lasts longer than the FBGVNP. The main influencing factor lies in the dendrite extension operation. Because the neuron number is fixed in one TrueNorth core, when there more synapses need to be accumulated dendrite extension has to be applied, though that takes additional time and hardware resources.

**Figure 6:**
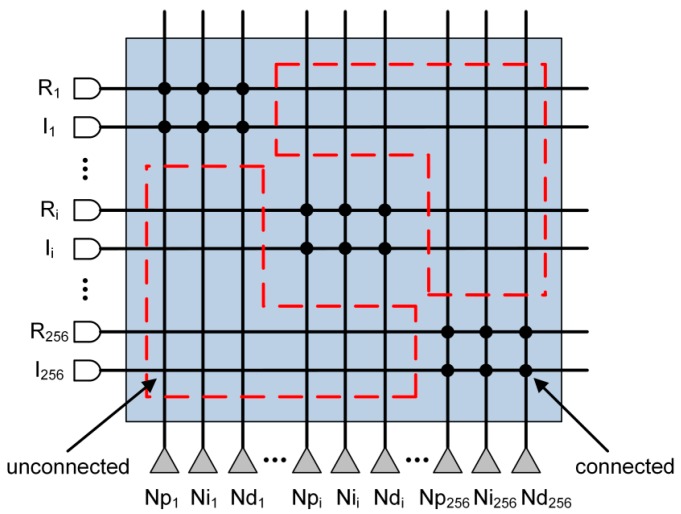
Connection diagram in TrueNorth of the second layer of the reference FCPIDNN.

On the other hand, when the number of the neurons connected in one TrueNorth core is less than 256, it will create waste in the unconnected crossbar (memory resources for saving synaptic weights). For instance, Figure 6 shows the connection condition of the second layer of the reference FCPIDNN. The valid connection is less than 1% (1/128). That is, over 99% of the crossbar resource is wasted.

Relatively, FBGVNP employs the methodologies of granularity variability and integrated computing and addressing. That makes the number of neurons in one core capably vary as needed without additional hardware resources, which helps to improve the utilization of the hardware resources and reduce time delays.

## 3. Experiments and Discussion

### 3.1. Experiment Setup

**Figure 7:**
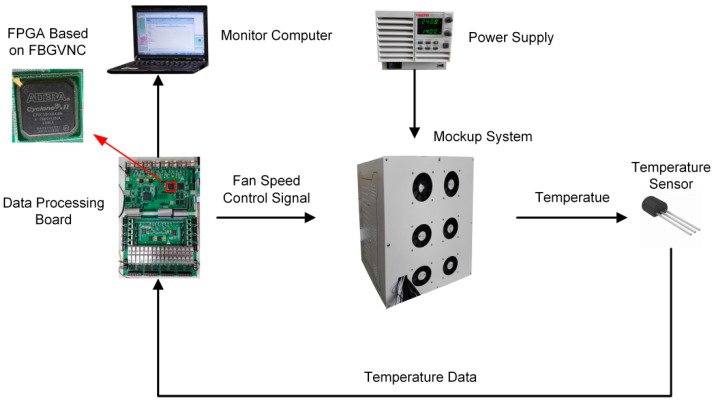
Structure of the temperature control system.

In this section, a FCPIDNN is mapped to the neuromorphic processor and applied in a temperature sensing and control system. The control system includes a mockup, a power supply, temperature sensors distributed in the mockup, a data monitoring computer, and a data processing circuit. The presented neuromorphic processor is realized in a FPGA placed on the data processing circuit. The structure of the temperature control system is shown in Figure 7.

**Figure 8:**
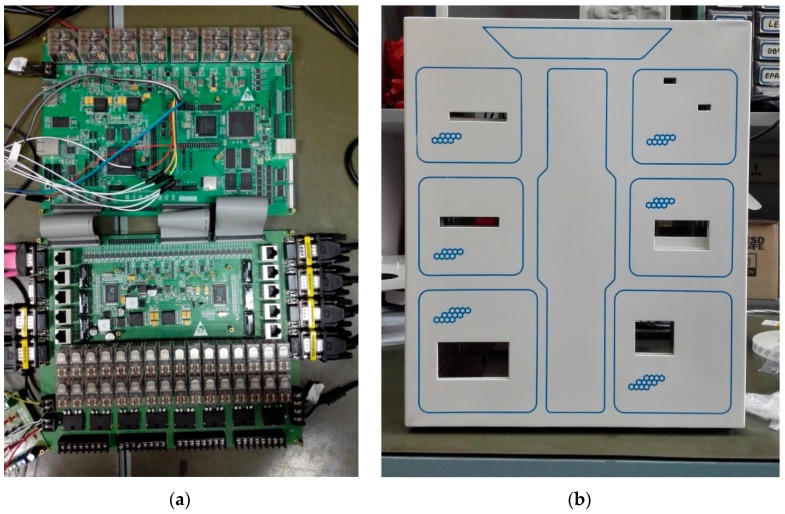
(**a**) Data processing printed circuit board (PCB); (**b**) Temperature sensing and control mockup.

The configuration of the mockup contains six fans indicated as Fan1 to Fan6 and six inside modules indicated as module 1 to module 6. The modules inside the mockup have different volumes and shapes. A heater for generating heat is placed inside each module. The six heaters have different levels of power. The temperature control actuators inside the mockup are cooling fans. The local temperatures around the six modules are detected by distributed temperature sensors. The data processing circuit acquires the temperatures from the sensors through the universal asynchronous receiver-transmitter (UART) port, runs the temperature controller, and outputs the pulse width modulation (PWM) control signals to the actuators. The data monitoring computer is used to collect the temperature information via the UART port from the data processing circuit [28]. The prototypes of the data processing printed circuit board (PCB) and the temperature sensing and control mockup are shown in Figure 8.

### 3.2. Experiment Results and Discussions

**Figure 9:**
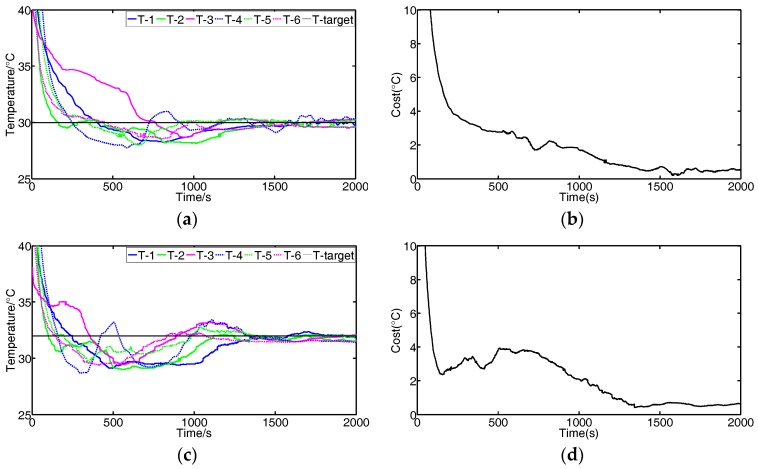
Experimental results of temperature control response when the target temperature is set to constant. (**a**,**c**) Temperature responses of different modules (target temperature is 30 °C and 32 °C, respectively); (**b**,**d**) changes of cost function values corresponding to the temperature responses shown in (**a**,**c**).

**Figure 10:**
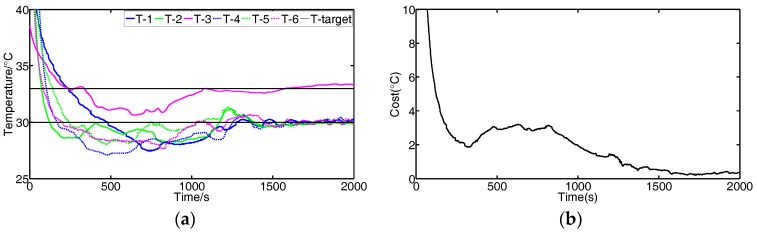

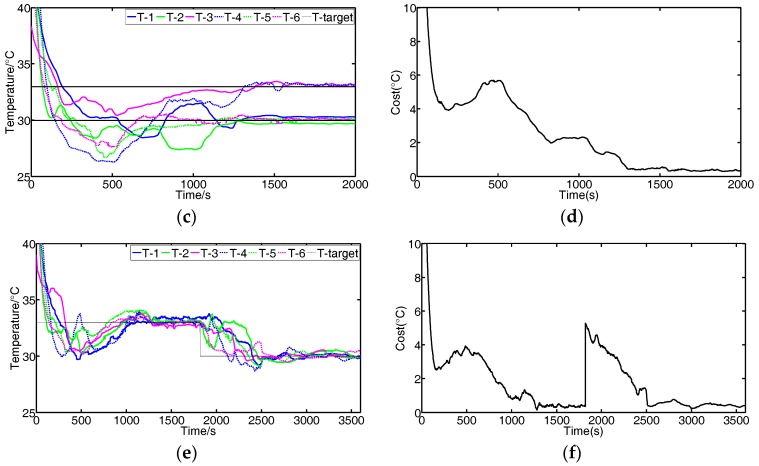
Temperature control scenarios. (**a**,**b**) Temperature target of module 3 is set to 33 °C and others are set to 30 °C. (**c**,**d**) Temperature target of modules 3 to 4 is set to 33 °C and others are set to 30 °C. (**e**,**f**) Temperature target is set to 33 °C at first 1800 s and then turned to 30 °C; (**b**,**d**,**f**) are the cost function values corresponding to (**a**,**c**,**e**).

We conduct experiments to validate the effectiveness of the FBGVNP-based temperature controller. Figure 9 shows the response process when the temperature targets for all modules are set to constant. Figure 10 shows the response process with multi-targets or the variational target.

In Figure 9, it indicates that the different modules’ temperatures approach the set target, and the differences between the actual and target temperatures decrease. The cost function value is formulated as the following Equation (1).

(1) $$J(n\;)=\sqrt{\frac{1}{2}\overset{6}{\underset{m=1}{\sum }}e_{m}^{2}(n)}$$

where the difference between the target and actual temperature is represented by *e*, the number of the module is represented by *m*, and the sample number is represented by *n*.

Figure 10 shows the results using the FBGVNP-based controller to deal with the temperature control with different targets, which further validates the effectiveness of the temperature control and exhibits its good robustness and adaptability to different scenarios. Figure 10a,b shows the control results when two different targets were set. In the process, the target temperature of module 3 was set to 33 °C and others were 30 °C. It can be seen that the cost function gradually reached the minimal and the modules’ temperatures approached their specific targets. Figure 10c,d shows the control results when the target temperature for modules 3 to 4 was set to 33 °C and the target temperature for other modules was 30 °C. The modules approached both their targets, respectively. Figure 10e,f show the control results if the modules’ targets varied, i.e., 33 °C at the beginning and then turned to 30 °C. The FBGVNP-based controller modulated the modules’ temperature to the set targets successfully and the cost functions got to minimal at last.

## 4. Conclusions

In this paper, we propose a FPGA-based granularity variable neuromorphic processor FBGVNP. The traits of FBGVNP can be summarized as granularity variability, scalability, integrated computing, and addressing ability: First, the number of the neurons is variable rather than constant in one core; second, the number of internal neural computing units and the scale of the multi-core network can be extended as needed; third, the neuron addressing and computing processes are executed simultaneously. Additionally, a comparison between the FBGVNP and an existing neurosynaptic chip TrueNorth is conducted. Moreover, a neural network-based controller is mapped to FBGVNP and applied to a multi-input, multi-output (MIMO), temperature-sensing and control system. Experiments validate the effectiveness of the presented neuromorphic processor. The FBGVNP provides a new scheme for building ANNs, which is flexible and highly energy-efficient, and can be widely applied in many areas with the support of the state-of-the-art algorithms in machine learning.

## Figures and Tables

**Table 1:** Comparison of neuromorphic processors and General Purpose Processors (GPPs) for implementing neural network.

Features	General Purpose Processors (GPPs)			Neuromorphic Processors	
	multicore CPU	GPGPU	MCU	ASIC-based	FPGA-based
computing process	sequential			parallel	
computing structure	centralized			distributed	
energy-efficiency	low			best	better
development round	short			longest	longer
cost	high	high	low	highest	moderate

**Table 2:** The format of the transmission data packet.

Dx	Dy	Destination Unit Index	Destination Cell Index	Neuron Output
8 bits	8 bits	4 bits	8 bits	8 bits

**Table 3:** Comparison of FBGVNP and TrueNorth.

Processors	Neuron Number	Computing and Addressing	Scalability	Power Consumption
FBGVNP	variable	integrated	internal & external	high
TrueNorth	fixed	separated	external	low

**Table 4:** Execution time comparison of FBGVNP and TrueNorth based on FCPIDNN.

Processors	1st Layer (Gclk Period)	2nd Layer (Gclk Period)	3rd Layer (Gclk Period)	4th Layer (Gclk Period)
FBGVNP	1	1	1	1
TrueNorth	1	1	1	2
